# Modelling science trustworthiness under publish or perish pressure

**DOI:** 10.1098/rsos.171511

**Published:** 2018-01-10

**Authors:** David Robert Grimes, Chris T. Bauch, John P. A. Ioannidis

**Affiliations:** 1 School of Mathematics and Physics, Queen’s University Belfast, BT7 1NN, UK; 2 Department of Oncology, University of Oxford, Old Road Campus, Oxford OX3 7DQ, UK; 3 Department of Applied Mathematics, University of Waterloo, 200 University Avenue W, Waterloo, Ontario, Canada N2L 3G1; 4 Meta-Research Innovation Center at Stanford (METRICS), Stanford University, SPRC, MSOB X306, 1265 Welch Road, Stanford, CA 94305, USA; 5 Department of Medicine, Stanford University, SPRC, MSOB X306, 1265 Welch Road, Stanford, CA 94305, USA; 6 Department of Health Research and Policy, Stanford University, SPRC, MSOB X306, 1265 Welch Road, Stanford, CA 94305, USA; 7 Department of Biomedical Data Science, Stanford University, SPRC, MSOB X306, 1265 Welch Road, Stanford, CA 94305, USA; 8 Department of Statistics, Stanford University, SPRC, MSOB X306, 1265 Welch Road, Stanford, CA 94305, USA

**Keywords:** research ethics, research fraud, science trustworthiness, public trust in science, publish or perish

## Abstract

Scientific publication is immensely important to the scientific endeavour. There is, however, concern that rewarding scientists chiefly on publication creates a perverse incentive, allowing careless and fraudulent conduct to thrive, compounded by the predisposition of top-tier journals towards novel, positive findings rather than investigations confirming null hypothesis. This potentially compounds a reproducibility crisis in several fields, and risks undermining science and public trust in scientific findings. To date, there has been comparatively little modelling on factors that influence science trustworthiness, despite the importance of quantifying the problem. We present a simple phenomenological model with cohorts of diligent, careless and unethical scientists, with funding allocated by published outputs. This analysis suggests that trustworthiness of published science in a given field is influenced by false positive rate, and pressures for positive results. We find decreasing available funding has negative consequences for resulting trustworthiness, and examine strategies to combat propagation of irreproducible science.

## 1. Introduction

In academia, the phrase ‘publish or perish’ is more than a pithy witticism—it reflects the reality that researchers are under immense pressure to continuously produce outputs, with career advancement dependent upon them [1]; [2]. Academic publications are deemed a proxy for scientific productivity and ability, and with an increasing number of scientists competing for funding, the previous decades have seen an explosion in the rate of scientific publishing [3]. Yet while output has increased dramatically, increasing publication volume does not imply that the average trustworthiness of publications has improved. As science pivots on replicable findings, for the purposes of this work we define a trustworthy finding as one that can be replicated independently, corresponding to a true positive or negative. A previous paper by Ioannidis [4] has outlined the reasons why many published research findings are false, and the dubious use of *p*-values for significance in research findings has of late been widely discussed [5–9]. Across much of experimental science from psychology [10] to biomedical science [11–13] and cancer research [14], there is concern over an apparent reproducibility crisis.

Despite their vital importance in conveying accurate science, top-tier journals possess a limited number of publication slots and are thus overwhelmingly weighted towards publishing only novel or significant results. Despite the fact that null results and replications are important scientific contributions, the reality is that journals do not much care for these findings. Researchers are not rewarded for submitting these findings nor for correcting the scientific record, as high-profile examples attest [15,16]. This pressure to produce positive results may function as a perverse incentive. Edwards & Roy [17] argue that such incentives encourage a cascade of questionable findings and false positives. Heightened pressure on academics has created an environment where ‘Work must be rushed out to minimize the danger of being scooped’ [18]. The range of questionable behaviour itself is wide [19]. Classic ‘fraud’ (falsification, fabrication and plagiarism (FFP) [20]) may be far less important than more subtle questionable research practices, which might include selective reporting of (dependent) variables, failure to disclose experimental conditions and unreported data exclusions [21].

So how common are such practices? A study of National Institute of Health (NIH)-funded early and mid-career scientists (*n*=3247) found that within the previous 3 years, 0.3% admitted to falsification of data, 6% to a failure to present conflicting evidence and a worrying 15.5% to changing of study design, methodology or results in response to funder pressure [22]. An overview by Fanelli [23] has shown that questionable research practices are as common as 75%, while fraud *per se* occurs only in 1–3% of scientists. These findings are alarming, yet quantification of these perverse incentives is vital if we are to understand the potential extent of the underlying problem, and formulate strategies to address it. This is an underdeveloped area, but one which is slowly growing—recent works by Smaldino & McElreath [24,25] have employed elegant dynamic models to demonstrate that even when there is no attempts at fraud or untoward research practices, selection based solely on published output tends to produce poorer methods and higher false discovery rates, a phenomenon they term ‘the natural selection of bad science’.

Suboptimal science and fraud can take myriad forms which renders it difficult to detect [26]. For the purposes of this article, we define fraud as an explicit ‘intention to deceive’ [27]. A more recent investigation [23] put the weighted mean percentage of scientists committing research fraud as high as 1.97%, with over a third admitting to questionable research practices. The same investigation found that about 14.12% of scientists reported observing fraudulent research behaviour in colleagues. Another study [28] found that 5% of responding authors claimed to be personally aware of fabricated or misrepresented data in a trial they had participated in. A study of bio-statisticians [29] found that over half of respondents reported being aware of research misconduct.

A 2012 [30] analysis found that FFP offenses rather than honest error accounted for 67.4% of retracted publications, with the rate of retraction due to fraud increasing 10-fold since 1975. An important question is whether scientists who are unethical (fraudulent) or sloppy (careless) may thrive and even outperform diligent scientists in a system driven by publish or perish pressure. As it is impossible to identify all unethical and careless scientists, one can perform mathematical modelling of science under different assumptions and find out how these scientists fare and what the implications are for the overall trustworthiness of science.

To better understand the impact of publish or perish on scientific research, and to garner insight into what practices drive the trustworthiness of published science is of paramount importance if we are to counteract any detrimental impacts of such practices. In this work, we present a simple but instructive model of scientific publishing trustworthiness under the assumption that researchers are rewarded for their published output, taking account of field-specific differences and the proportion of resources allocated with funding cycle. The factors that influence resultant trustworthiness in simulation are quantified and discussed, as well as implications for improving the trustworthiness of scientific publishing. It is important to note that the motivations of scientists and ecosystem of scientific publishing are inherently complex, and we do not expect the model discussed to be deterministically predictive—rather, the model is presented as a tool for allowing us to formulate publication dynamics in a population of scientists in a formal and precise way, subject to certain caveats.

## 2. Model outline

### 2.1. Basic model and assumptions

To construct a simple model of publication rewards, we define the total amount of available funding for research as *R*(*t*). Per unit of funding in a given field, there is a global discovery rate of *D*_*R*_, which includes a proportion *p*_*T*_ of positive/significant results, a proportion *p*_*F*_ of false positives, and a proportion *n* of null results. Null results in principle can include both true negatives and false negatives, but given the bias towards positive results we will not discriminate between these two in this investigation. The relative proportion of positives and nulls will be inherently field-specific—certain disciplines will be more prone to false positives, while others tend to yield less ambiguous results. As the quantities are proportions, we have that

2.1 $$p_{T}+p_{F}+n=1.$$

In certain fields, the false positive rate may be high, and so diligent researchers take measures to falsify positive results and test their results multiple times. Even when research groups are very diligent, they may occasionally happen upon a false or misleading result which is hard to eliminate and due to experimental or theoretical difficulty rather than carelessness. For the diligent cohort, this will be as low as can reasonably be achieved and so we state they submit a small fraction, *ϵ*, of their false positives for publication. Researchers exist on a spectrum, but for simplicity we may broadly sub-divide this spectrum into three distinct classes.

(i) *Diligent cohort*. This group take pains to replicate experiments and do not dishonestly manipulate results. Their false positive submission fraction is *ϵ*, thus as low as reasonably possible. They account for a fraction *f*_*D*_ of the initial total, and parameters relating to them have subscript D.
(ii) *Careless cohort*. This group do not falsify results, but are much less careful at eliminating spurious positive results. They may also have questionable practices that lead them to false positives. As a result, they have a false positive submission rate of *cϵ* where *c*>1. They account for a fraction *f*_*C*_ of the initial total, and parameters relating to them have subscript C.
(iii) *Unethical cohort*. This group appear broadly similar to the diligent group, but with one crucial difference in that they may occasionally manipulate data or knowingly submit dubious results at a rate of *δ* beyond global discovery rate. For convenience, instead of defining a higher value of *D*_*R*_ in this group to account for the higher ‘discovery’ rate, we retain the same parameter value of *D*_*R*_ for the unethical cohort but allow *p*_*T*_+*p*_*F*_+*n*+*δ*>1, so that their realized ‘discovery’ rate is higher than the other groups. They account for a fraction *f*_*U*_ of the initial total, and parameters relating to them have subscript U.

The funding held by the diligent cohort at a given time is *x*(*t*), with *y*(*t*) held by the careless cohort and *z*(*t*) by the unethical cohort, so that

2.2 x(t)+y(t)+z(t)=R(t).

With these assumptions, we can model the theoretical impact of a paradigm where researchers are rewarded with funding and success in direct relation to their publication output. As outlined in the Introduction, there is huge pressure on scientists to submit positive or ‘novel’ findings, while findings confirming the null hypothesis are frequently side-lined. Under such a selection pressure, all researchers will aim to submit their significant positive results for publication. The respective rates of submission per unit funding for the diligent, careless and unethical cohorts are accordingly

2.3 $$\left(\begin{array}{c} S_{D+}\\ S_{C+}\\ S_{U+} \end{array}\right)=D_{R}\;\left(\begin{array}{c} p_{T}+\epsilon p_{F}\\ p_{T}+c\epsilon p_{F}\\ p_{T}+\epsilon p_{F}+\delta \end{array}\right)$$

The rate at which null results are submitted is less clear—in general, there is a significant bias in publication towards significant results. As a consequence, negative findings are often shunned by high-impact journals, and scientists are disinclined to submit them, meaning that potentially important null results may not ever see the light of publication, the so-called ‘file drawer’ problem. We assume that each cohort submit only a fraction of their null results in the proportions *β*_D_,*β*_C_,*β*_U_ such that

2.4 $$\left(\begin{array}{c} S_{D-}\\ S_{C-}\\ S_{U-} \end{array}\right)=D_{R}n\;\left(\begin{array}{c} \beta_{D}\\ \beta_{C}\\ \beta_{U} \end{array}\right).$$

Equations (2.1)–(2.4) comprise the researcher-specific parameters, and we must further quantify the journal-specific elements also. Competition for space in field-specific top-tier journals is fierce, and we denote the combined carrying-capacity of these field-specific top-tier journals as *J*(*t*). These journals exhibit a clear bias towards positive results, with a positive-publication weighing fraction of published articles, *B*, describing significant results. Thus, presuming that more submissions are obtained than can be published, we can quantify the probability that a positive result (*ν*_*P*_(*t*)) or a negative result (*ν*_*N*_(*t*)) is published. These probabilities are given by

2.5 $$\left(\begin{array}{c} \nu_{P}(t)\\ \nu_{N}(t) \end{array}\right)=\left(\begin{array}{c} \frac{JB}{x(t)S_{D+}+y(t)S_{C+}+z(t)S_{U+}}\\ \frac{J(1-B)}{x(t)S_{D-}+y(t)S_{C-}+z(t)S_{U-}} \end{array}\right).$$

From this, we can then yield an expression for the publication rate per unit of funding for the diligent, careless and unethical cohorts, which are, respectively,

2.6 $$\left(\begin{array}{c} L_{D}(t)\\ L_{C}(t)\\ L_{U}(t) \end{array}\right)=\nu_{P}(t)\;\left(\begin{array}{c} S_{D+}\\ S_{C+}\\ S_{U+} \end{array}\right)+\nu_{N}(t)\;\left(\begin{array}{c} S_{D-}\\ S_{C-}\\ S_{U-} \end{array}\right).$$

The average rate of publications per unit of funding per unit time in top-tier journals for a given field is thus

2.7 $$A(t)=\frac{J}{x(t)+y(t)+z(t)}.$$

If researchers are rewarded with funding based solely on their published output, we can quantify the impact of this with time by employing a recursive series solution at discrete time steps, corresponding to funding cycles. If funding is allocated to each cohort based upon their output at the beginning of the previous funding cycling, and we assume total funding remains constant (*dR*/*dt*=0) then the funding available for each cohort at each successive time step is

2.8 $$\left(\begin{array}{c} x(t+1)\\ y(t+1)\\ z(t+1) \end{array}\right)=\left(\begin{array}{c} \frac{L_{D}(t)}{A(t)}x(t)\\ \frac{L_{C}(t)}{A(t)}y(t)\\ \frac{L_{U}(t)}{A(t)}z(t) \end{array}\right).$$

### 2.2. Variable funding resources

We also consider the fact that the total amount of funding may not remain constant, so we may model the impact of changing funding scenarios. For simplicity, we assume it changes at some constant rate *G*, which can be negative (for diminishing funding, the likes of which might occur with a decrease in NIH or EU funding budgets), zero (for constant funding, as in equation (2.8)) or positive (increasing funding). New funding is allocated at random in proportions reflecting the typical make-up of new researchers, and accordingly the refined equations are

2.9 $$\left(\begin{array}{c} x(t+1)\\ y(t+1)\\ z(t+1) \end{array}\right)=\left(\begin{array}{c} \frac{L_{D}(t)}{A(t)}x(t)+f_{D}G\\ \frac{L_{C}(t)}{A(t)}y(t)+f_{C}G\\ \frac{L_{U}(t)}{A(t)}z(t)+f_{U}G \end{array}\right).$$

### 2.3. Research fraud detection

For unethical researchers, we can look at a slightly more complicated scenario where dubious publications have a probability of detection leading to denial of funding, *η*. We further assume this penalization only applies to dubious results which were published rather than just submitted. If this consideration is taken into account, then we modify the last part of equation (2.9) to reflect this so that

2.10 $$z(t+1)=\left(\frac{L_{U}(t)}{A(t)}-D_{R}\eta \delta \nu_{p}(t)\right)\;z(t)+f_{U}G.$$

### 2.4. Rewarding diligence

The diligent cohort have intrinsically lower submission rates than other groups, and consequently are more likely to suffer under a publish or perish regime, despite the importance of their reproducible work. To counteract this, it has been suggested that rewarding diligence might counteract this trend [31,32]. We might envision an ideal situation where scientific works are audited for reproducibility by independent bodies, with groups who keep their reproducibility high and error rates below a certain unavoidable threshold (given by *D*_*R*_*ν*_*P*_*ϵ*) garnering a reward of *R*_W_. This in practice could only be achieved by the diligent cohort, and in the most simple case, their funding resources are given by

2.11 $$x(t+1)=\frac{L_{D}(t)}{A(t)}x(t)+f_{D}G+R_{W}.$$

### 2.5. Counteracting publication bias

It is also possible to envision a situation where journals do not give any preference to positive results over null results. In this case, we would expect researchers to submit all their results so that *β*_D_=*β*_C_=*β*_U_=1. In this case, *ν*_*P*_ and *ν*_*N*_ are replaced by a single function of time *ν*, given by

2.12 $$\nu (t)=\frac{J}{x(t)(S_{D+}+S_{D-})+y(t)(S_{C+}+S_{C-})+z(t)(S_{U+}+S_{U-})}.$$

### 2.6. Trustworthiness of published science

Finally, we define a metric for the trustworthiness of published science, defined as the proportion of reproducible results, *T*(*t*). This is given by

2.13 $$T(t)=1-\frac{\nu_{p}D_{R}(x(\epsilon p_{F})+y(c\epsilon p_{F})+z(\epsilon p_{F}+\delta ))}{J},$$

where the time arguments of *x*,*y*,*z* and *ν*_*p*_ have been excluded for clarity.

### 2.7. Parameter estimation and assumptions

To simulate the trends that would occur under these assumptions requires that we select appropriate parameters (these are detailed in table 1), which are used in all simulations unless otherwise stated in the text. It can be seen through inspection that discovery rate per unit resource *D*_*R*_ cancels in the analysis for *x*(*t*), *y*(*t*), *z*(*t*) and *T*(*t*), and accordingly this can be ascribed any real positive value without skewing analysis. When there is no fraud detection funding penalization (*η*=0), journal carrying capacity *J* also cancels in the analysis and does not impact results. Initially we assume also that *G*=0 so that funding levels remain constant.

**Table 1.** Parameters for initial simulations. Values in this table comprise the default initial assumptions, which are varied to investigate different conditions, as outlined in the respective relevant section.

parameter	value
journal carrying capacity (*J*)	120/cycle
total discovery rate per unit funding (*D*_*R*_)	15
initial proportion diligent researchers (*f*_*D*_)	0.65 [23]
initial proportion careless researchers (*f*_*C*_)	0.33 [23]
initial proportion unethical researchers (*f*_*U*_)	0.02 [23]
reasonable error rate (*ϵ*)	0.05 [9]
fraudulent submission rate per unit resource (*δ*)	0.0574
positive publication bias (*B*)	0.9
multiplicative factor for careless cohort—(*c*)	2
null/negative submission rates—(*β*_D_/*β*_C_/*β*_U_)	0.40
resource growth rate—(*G*)	0
fraud detection proportion—(*η*)	0
field-specific true positive fraction (*p*_*T*_)	0.2
field-specific false positive fraction (*p*_*F*_)	0.2

Estimation of fraudulent submission fraction per unit resource *δ* requires some elaboration, as this is notoriously difficult to ascertain and field-specific. A 1996 analysis by Fuchs & Westervelt [33] extrapolated from known cases to estimate that approximately 0.01% of published papers were fraudulent, though this is considered exceptionally conservative [27]. Empirical estimates of plagiarism vary markedly from 0.02 to 25% of all publications [26]. The frequency of paper retractions from the PubMed database for misconduct is about 0.02%, suggesting that fraud might be present in 0.02–0.2% of papers therein [34]. An investigation in the *Journal of Cell Biology* found inappropriate image-manipulation occurring in 1% of papers [35]. More alarmingly perhaps, a 1992 data audit by the United States Food and Drug Administration found deficiencies a in 10–20% of studies published between 1977 and 1990, with 2% of investigators deemed guilty of severe scientific misconduct [23,36,37].

For the purposes of this work, we will assume that FFP violations are present in 1% of the published literature so that *J*_*F*_=*J*/100. Defining *x*_*o*_=*x*(0), *y*_*o*_=*y*(0), *z*_*o*_=*z*(0) and *ν*_*p*_*O*__=*ν*_*p*_(0), then for any selected values of *D*_*R*_ and *J*, we can readily define the required rate by

2.14 $$\delta =\frac{J_{F}(x_{o}S_{D+}+y_{o}S_{C+}+z_{o}D_{R}(p_{T}+\epsilon p_{F}))}{z_{o}D_{R}(BJ-J_{F})}.$$

This is dependent on the true/false positive of the field, and we initially take an optimistic assumption that the 1% of published fraud occurs in fields with high levels of false positives, and will be less in fields with less ambiguity in results, so that the same value of *δ* is used for all simulations. This is calculated assuming *p*_*F*_=0.32 and *p*_*T*_=0.08 so that *δ*=0.057 as per table 1. The reasonable error rate is taken from *p*-value for significance, as discussed in Colquhoun [9]. Strictly speaking, Prof. Colquhoun puts forward an eloquent argument in the cited work that *p*<0.05 is a frequently abused metric, leading to false positives. For simplicity, however, we will presume that *ϵ*=0.05 reflects best reasonable practice in this simulation.

## 3. Results

### 3.1. Impact of the field-specific false positive rate

**Figure 1.**
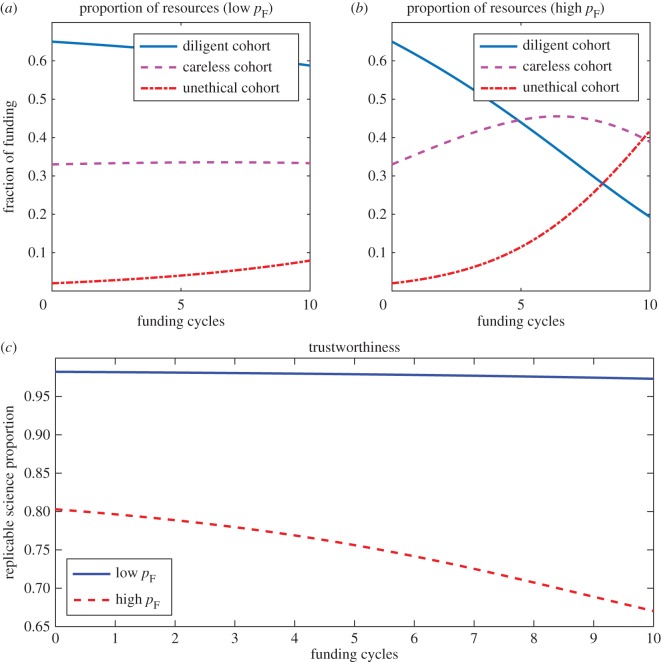
The impact of field-specific false positive rate on resources allocated and science trustworthiness. Panel (*a*) depicts the projected funding allocations in a field where false positives are relatively rare (*p*_*T*_=0.32, *p*_*F*_=0.08). By contrast, (*b*) shows the impact on resources consumed when false positives are the norm (*p*_*T*_= 0.08, *p*_*F*_=0.32). In (*c*), the trustworthiness (proportion of reproducible science) for both scenarios are depicted, indicating false positive rate highly drives the trustworthiness of scientific publication in a given field.

Figure 1 shows the change in funding proportions with time for each cohort in a field with a low rate of false positives (*p*_*F*_/(*p*_*T*_+*p*_*F*_)=0.25) and a field with a high rate of false positives (*p*_*F*_/(*p*_*T*_+*p*_*F*_)=0.8). What is immediately evident is that in fields where false positives comprise the bulk of positive results, the trustworthiness of published science suffers markedly, and careless and unethical cohorts are disproportionately rewarded at the expense of diligent researchers. Simulation results suggests that the trustworthiness of published science in any given field is strongly dependent on the false positive rate in that field under a publish or perish paradigm. As evidenced by the figure, outputs from diligent research and the number of trustworthy results still decline even when FPR is low with publish or perish incentives, as suggested by Smaldino & McElreath [24].

### 3.2. Impact of funding growth rate

**Figure 2.**
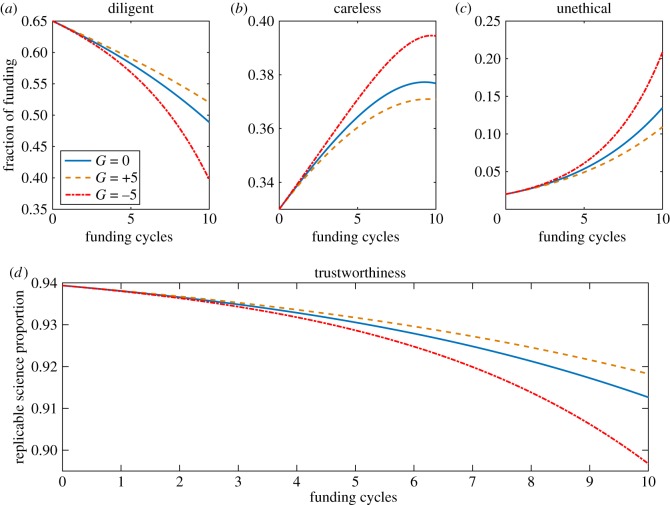
The impact of funding increases and decreases on funding allocation and science trustworthiness. Panels (*a*–*c*) depict the absolute proportion of funding resources allocated to diligent, careless and unethical cohorts, respectively, when funding changes at rates of 0, 5 and −5 per cycle.

As depicted in figure 2. The increasing of available funds (*G*) has the net effect of reducing publication pressure by bring down the average number of publications expected per unit funding, provided journal capacity stays roughly constant, reducing the likelihood of dubious publications being selected. Conversely, reducing funding increases the publication pressure and results in increased selection of suspect works and a fall in scientific reproducibility. The implications of this require some elaboration and are considered in the Discussion section.

### 3.3. Impact of increased fraud detection

**Figure 3.**
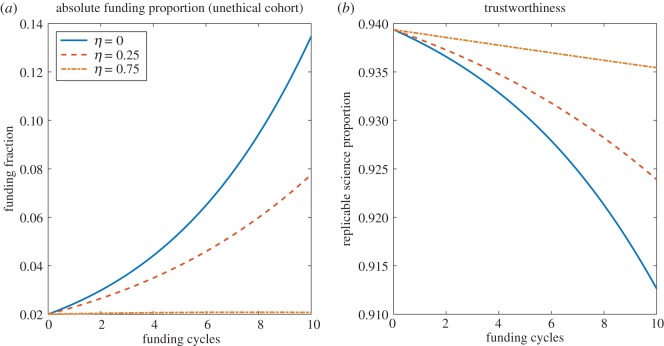
The impact of strict fraud detection/penalization. (*a*) Increasing the rate at which fraud is detected limits the amount of resources garnered by unethical cohorts, but (*b*) high values of *η* are required to markedly improve the trustworthiness of published science.

Figure 3 depicts the impact of aggressive fraud detection (*η*) and punishment. Increased fraud detection seems to improve science trustworthiness, but *η* has to be very high in practice to have a substantial impact on the proportion of funding allocated to unethical cohorts. Negating growth, the funding allocation to this group would only be expected to decrease with time if

3.1 $$\eta >\frac{1}{D_{R}\delta \nu_{P}}\left(\frac{L_{U}}{A}-1\right).$$

In practice, this is quite high, and for the values in table 1, a value of *η*>0.7688 would be required to fully diminish funding to this cohort in time.

### 3.4. Impact of rewarding diligence

By inspection, it is straightforward to show that for the amount of funding held by the diligent cohort to stay the same or increase, then the condition on *R*_W_ is

**Figure 4.**
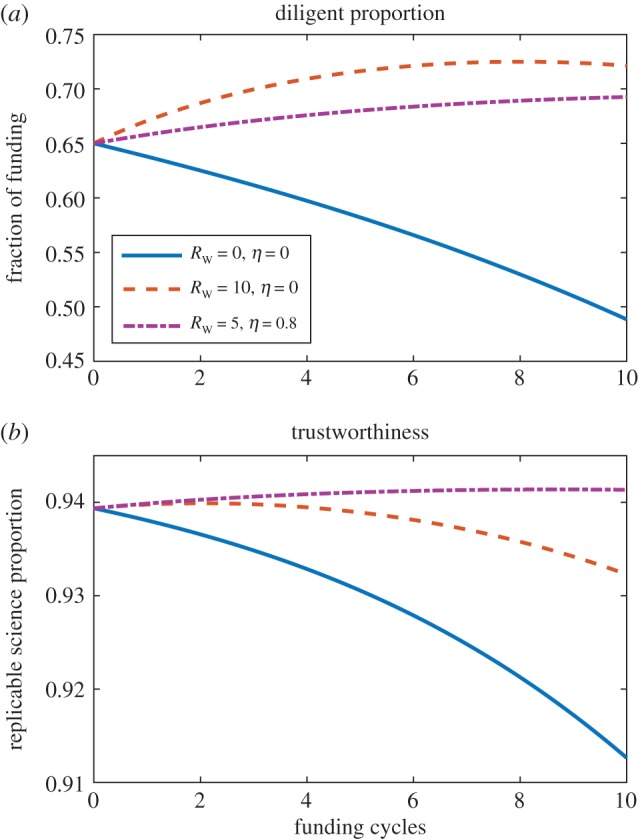
The impact of rewarding researcher for diligence. This improves the proportion of funding allocated to diligent researchers, but to improve science trustworthiness still requires non-zero values of *η* under this schema.

3.2 $$R_{W}\geq \left(1-\frac{L_{D}(t)}{A(t)}\right)\;x(t)-f_{D}G,$$

though in practice for most situations, *R*_W_ will have to be much greater than this minimum value. For the example depicted in figure 4, a large reward for diligence (*R*_W_=10) substantially increases the funds awarded to the diligent cohort. However, reproducibility still falls slowly if the unethical cohort is not removed. It is possible to both reward diligence and punish fraud, which can improve trustworthiness, as illustrated in figure 4.

### 3.5. Impact of the positive publication weighing

**Figure 5.**
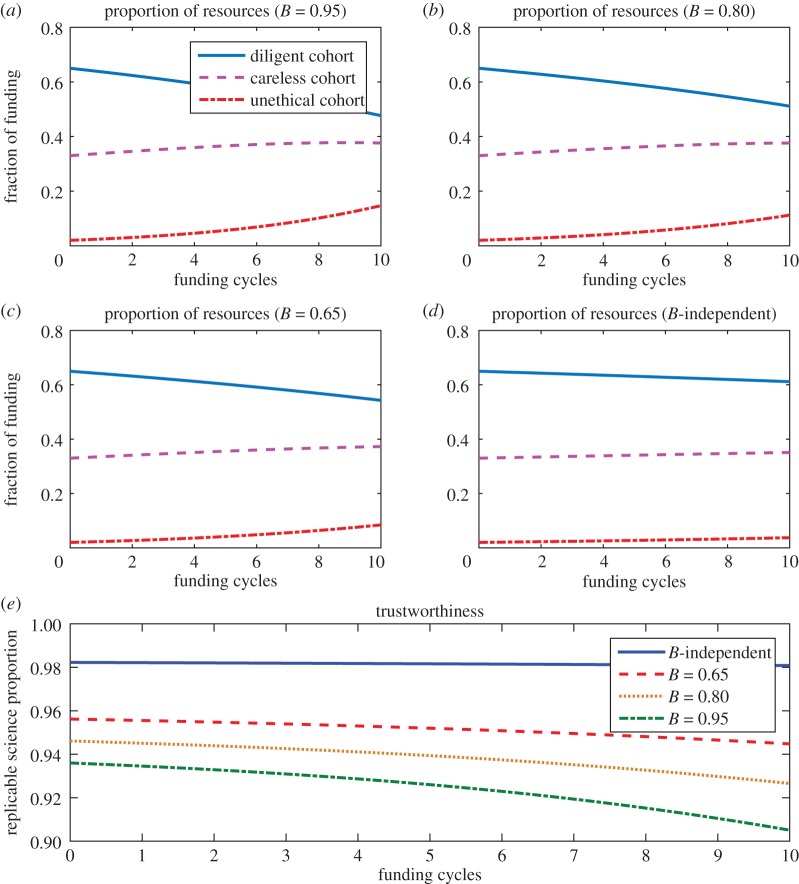
The impact of positive publication weighing on the trustworthiness of published science. (*a*–*c*) Show funding allocation 95%, 80%, 65% of published results are positive, respectively, while (*d*) depicts the situation when publications are completely agnostic. Science trustworthiness of all these scenarios in shown in figure (*e*), suggesting best trustworthiness obtained when journals were completely agnostic to whether a result was positive or null.

To simulate how published science fares under the rather artificial fixation of top-tier journals with positive novel results, figure 5 depicts how funding is allocated and science trustworthiness changes with varying values for *B*. In this simulation, *β*_D_=*β*_C_=*β*_D_=0.5 when publications were *B*≫0.5 (skewed towards positive publication). For the scenario where null and positive results were equally likely to be published, null results were as likely to be published so all were submitted and thus the fraction of null results submitted, respectively, were *β*_D_=*β*_C_=*β*_D_=1. Higher values of *B* lead to perverse rewarding of false positives and fraudulent results at the expense of diligent science. Best outcome for science trustworthiness was observed when journals were simulated as completely agnostic to findings.

### 3.6. Impact of initial unethical funding proportion

**Figure 6.**
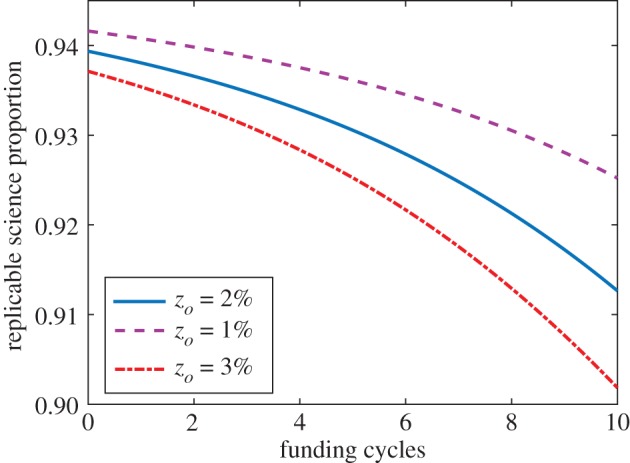
The trustworthiness of science in a field where *p*_*T*_=*p*_*F*_=0.2, with varying values of *z*_*O*_—the less funds are initially allocated to unethical cohorts, the better the resultant science trustworthiness.

Figure 6 depicts the sensitivity of trustworthiness to different assumptions of initial unethical funding proportion *z*_*o*_. As might be expected, increasing *z*_*o*_ has negative implications for published trustworthiness.

## 4. Discussion

The model presented is a simplification of a complex ecosystem, but gives some insight into what factors shape scientific trustworthiness. The model suggests that a fixation in top-tier journals on significant or positive findings tends to drive trustworthiness of published science down, and is more likely to select for false positives and fraudulent results. In our simulations, best outcome was obtained by simply paying no heed to whether a result was significant or not. This is akin to the model used by many emerging open access peer-reviewed journals such as *PLoS ONE*, who have a policy of accepting any work provided it is scientifically rigorous. Our simulation suggests this model of publishing should improve science trustworthiness, and it is encouraging that many other publishers are taking this approach too, including *Royal Society Open Science* and *Nature Scientific Reports*. As of 2017, *Scientific Reports* has surpassed *PLoS ONE* as the world’s biggest mega-journal [38]. However, there is an important point to consider in the form of the parameter *J* (the publication carrying-capacity). This can be highly field-specific, comprising the top-tier journals in that specific field. In general, these publications are focused on prestige rather than rapid dissemination of science and it is unlikely these journals would move to replicate the approach of rapid open-access publishers. Accordingly, the suggestion that top-tier journals might aspire to treat all studies, regardless of their results, as equally worthy of publication is likely to be an unworkable ideal.

Indeed, there is still a perception that such journals are for ‘trivial’ or unimportant results, and that positive or important results should still go to a few journals with extreme competition for space. Empirical evaluations show that small studies published in top-impact journals have markedly exaggerated results on average compared with similar studies on the same questions published in journals of lesser impact factor [39]. This suggests that the pressure to publish in these flagship journals may still be very real, despite the option of publishing in less competitive journals. The analysis here suggests that science trustworthiness is affected too by changes in funding resources, and that when an increase of funding improves the overall trustworthiness of science, as depicted in figure 2. Conversely when this is diminished, the increased competition on scientists appears to create conditions when false positives and dubious results are more likely to be selected for and rewarded. This is a natural consequence of the model, but requires careful interpretation. Crucially, it is important to note that there is no mechanism in the model for unethical or careless researchers to transition into diligent scientists. Rather, decreasing funding increases competition, and amplifies the career advantages of questionable findings. Conversely, if global funding rates are increased, then competition for resources decreases and the advantage of suspect findings is somewhat mitigated. While beyond the scope of this work, such a prediction could be empirically tested by analysing situations when research budgets change markedly, such as the doubling of the NIH budget from 1998 to 2003.

The model presented pivots on the assumption of a scenario that publication is the dominant metric upon which scientists are rewarded, and elucidates the potential consequences of such a situation. It is important to note this is a substantial simplification, and there are other metrics by which scientists are assessed, including other measures of impact, awards and citations. However, the number of publications attributed to a scientist has a marked effect on their career success, with more publications associated with principal investigator status, and acquisition of funding [40]. The average number of authors per paper is increasing over time, and this is not just due to more interdisciplinary work, but also due to a greater demand for having more papers in one’s CV [41]. The model also implicitly assumes that output is an approximately linear function of funding in a given field. The exact applicability of this assumption may vary across fields. For example, wet-lab sciences require a certain threshold of continuous funding just to operate, whereas computational or theoretical sciences may be able to operate with comparatively little funding. Presuming direct comparison of researchers and their teams across a given field, however, the assumption of direct correspondence between resources and outputs is reasonable, although outliers are to be expected.

One curious result persistently seen in the model was that diligent researchers are unfairly affected by careless or unethical conduct, with avoidable false positives or unethical publications garnering disproportionate reward at their expense. Simply increasing fraud detection does not do much to stop this, as careless researchers benefit from the gap in the market, out-producing their diligent colleagues, as shown in figure 3. This appears to be an unfortunate and seemingly unavoidable consequence of a ‘publish or perish’ system. However, in good scientific environments carelessness would be sooner or later detected and potentially penalized. We can estimate how much of a penalty for carelessness or reward for diligence we need so as to inverse the worsening trends that we observe, by manipulating equations similar to the manner outlined for unethical conduct. However, this approach risks being ruthlessly punitive, punishing honest mistakes with the same severity reserved for the most egregious abuses of scientific trust.

While a penalty for carelessness has intuitive appeal, distinguishing between honest and careless errors is fraught with difficulty. As has been argued elsewhere [31,32], rewarding diligence is perhaps a better way to ensure researchers do not suffer for good conduct. A simple model of this is shown in figure 4, and indeed this suggests rewarding diligence improves the proportion of funding allocated to diligent groups. However, it requires some penalty for bad conduct to keep unethical cohorts from benefiting at the expense of others. In practice, this level of detection appears to have to be relatively high, which of course would require considerable resources to achieve. It should be noted too that the false positive rate of a field has a significant impact on science trustworthiness, as illustrated in figure 1. A high type II error rate provides ample cover for the small minority of unethical researchers to cheat without overt fear of detection [23,27], perhaps explaining the elevated prevalence of dubious practice in biomedical science [23], in particular.

The model presented is a much simplified picture of reality, but it allows us to examine how different factors might influence the trustworthiness of published science, and potentially suggest strategies to improve it. As the motivations of and pressures on scientists are incredibly complex, it is important to recognize the limitations in the model too. The three cohorts presented here would in reality constitute a spectrum. The sub-divisions in this work are relatively arbitrary and informed by the available data on researcher populations, though it would be easily possible to extend this to consider more subpopulations if desired. Scientific conduct is notoriously difficult to quantify, and the assumptions we have used in this work reflect the best estimates to date [23].

We can also envision a situation where authors are awarded solely on the basis of positive findings, so that negative findings have no funding benefit. We can apply the model to these circumstances too, with the realization that under such a scheme, there would be no incentive for authors to submit negative results. In this case, *B*=1 and all *β* terms reduce to zero. Essentially then, one gets a similar result to the one shown in figure 5*a*, with even further reduction in trustworthiness. Finally, measures that can be adopted to begin changing the culture of fixation on novel positive results include the establishment of awards by academic societies designed to recognize methodological rigour rather than positive results, as well as the explicit recognition of material published in online repositories as relevant material in university tenure and promotion guidelines.

It is also worth considering how the positive publication weighing might impact on the ‘file-drawer’ problem [42]. This was the observation first articulated by Rosenthal in 1979 that researchers tended to not invest their energy trying to publish null findings, instead burying them in a file-drawer. The great tragedy of this is that essential null results are often disregarded by the scientists who discover them, meaning others labour down fruitless avenues. In the model, we have implicitly assumed a version of this by assuming researchers submit only a portion of their negative findings (*β*) for consideration. It would be useful to know precisely how much is never submitted, and to gauge the extent of the file-drawer problem. Certainly estimates have been made in some fields, notably by Franco *et al*. [43], who determined that in one study of publications in social sciences, only 35% of the null results were ever written up (in good agreement with our estimates for *β* in table 1) and ultimately, just over 20.8% of these findings were published. Also for NIH-funded clinical trials, 32% remained unpublished a median of 51 months after their completion [44]. Whether these patterns apply also in other fields remains to be seen. One approach might be to consider the issue from an energy-expenditure point of view or game-theory approach which could be coupled with the model to estimate how much vital science never reaches the public domain, though this is beyond the scope of this investigation.

A more sophisticated future analysis might include variables that respond to the available funding. For example, the fraudulent publication rate *δ* is treated as a constant in this work for the most part, but it is easy to imagine a situation where this increases with shrinking funding, or where the number of investigators willing to engage in such practices is a function of available funding. This is not considered here, but the model presented could be easily adapted to probe this further. Future work with more sophisticated models could explore how best to implement these and other possible interventions designed to improve science trustworthiness. For instance, trustworthiness as a function of positive publication bias (B) and fraud detection rate (*η*) could be computed and optimization approaches could be applied to determine the optimal combination of B and *η* to improve science trustworthiness. These parameters can be somewhat influenced by large academic societies, government agencies or independent foundations for instance, who could fund efforts to detect fraud in published work and support research concerning null results.

It is also important to note that the model results pivot explicitly on the assumption that scientists are forced to operate under a ‘publish or perish’ regime, and rewarded solely on output. Thus, there is another way to improve the trustworthiness of published science—while publications are indeed one measure of productivity, they are not necessarily the sole measure. While a much harder aspect to gauge, trustworthiness is more fundamentally important. For their part, scientific journals should realize that issues such as replication and null findings are equally vital to good science as eye-catching ‘new’ results. This is slowly beginning to be recognized, with some groups coming to the forefront of championing reproducible research methods [45]. The consequences detailed in this manuscript only arise when publishing quantity is the dominant measure of an academic’s worth, but in reality this should only be one consideration among many. The model suggests that if publishing is the sole criteria under which academics are judged, then dubious conduct can thrive.

We accordingly need to address alternative ways to assess researchers, and to encourage judicious diligence over dubious publishing. The model outlined here is far from complete, but yields some insights into the factors that shape the trustworthiness of published science. There is already evidence that pressure to publish is driving researcher burn-out and cynicism in published research [46], negatively affecting both research and the researchers themselves [47,48]. Other studies have not found a clear association of some productivity incentives with bias [49], but these incentives may be confounded in that sometimes they coexist with other features and research practices that tend to increase also quality of research, rather than just quantity of publications. Crucially, bogus findings risk undermining public confidence in science. Among notable examples [50–52], the fraudulent *Lancet* MMR-Autism paper [53] is especially infamous, remaining a cornerstone of anti-vaccine narratives [54].

Scientific publishing is not intrinsically flawed, and complete, unbiased publication is essential for scientific progress. This work illuminates potential consequences of a system where publication is the dominating measure of academic success, and strongly suggests we should consider the consequences of our incentives, and look at changing how academics are evaluated. This is key not only to appreciating the exceptional pressures wrought upon researchers by a strict publish or perish imposition, but to improving science itself. This would not only benefit those working in the field, but is crucial if public trust in science is to be maintained.

## Data accessibility

A demonstration version of the model coded is available at https://github.com/drg85/Publish-or-Perish, coded for MATLAB and as a windows stand-alone application.
